# Role of Pre-procedural MRI Parameters and Urodynamic Findings in Predicting Clinical Success After Prostate Artery Embolization

**DOI:** 10.7759/cureus.111178

**Published:** 2026-06-19

**Authors:** Zain Nayyer, Maryum Shafiq, Sadia Anwar, Nithin Thomas, Rohan Chandra, Georgia Lauren Mae Bailey, Mohammod Imran Hossain, Payal Subhash Joshi, Muhammad Hamid Chaudhary

**Affiliations:** 1 Diagnostic Radiology, Fatima Bashir Hospital, Gujranwala, PAK; 2 Urology, Worcestershire Acute Hospitals National Health Service (NHS) Trust, Redditch, GBR; 3 Diagnostic Radiology, Institute of Nuclear Medicine and Radiation Oncology (INMOL), Lahore, PAK; 4 Radiology, Worcestershire Acute Hospitals National Health Service (NHS) Trust, Worcester, GBR; 5 Radiology, University of Sheffield, Sheffield, GBR; 6 Cardiac Surgery, Chaudhry Pervaiz Elahi Institute of Cardiology, Multan, PAK

**Keywords:** functional urology, general radiology, prostate artery embolization, prostate artery embolization (pae), uro-radiology

## Abstract

Background

Prostate artery embolization (PAE) has emerged as a minimally invasive alternative for the management of lower urinary tract symptoms (LUTS) secondary to benign prostatic hyperplasia (BPH). However, variability in clinical outcomes highlights the need for reliable pre-procedural predictors. This study aimed to evaluate the role of pre-procedural magnetic resonance imaging (MRI) parameters and urodynamic findings in predicting clinical success following PAE.

Methods

This retrospective observational study included 76 patients with symptomatic BPH who underwent PAE. Baseline clinical variables, MRI parameters (prostate volume, transition zone index, intravesical prostatic protrusion, adenomatous-dominant hyperplasia), and urodynamic parameters (maximum flow rate (Qmax), detrusor pressure (Pdet)Qmax, bladder outlet obstruction index (BOOI)) were recorded. Clinical success was defined as a ≥25% reduction in International Prostate Symptom Score (IPSS) and/or an increase in Qmax of ≥2 mL/s at 3 months. Patients were categorized into successful and unsuccessful outcome groups. Statistical analysis included group comparisons and multivariate logistic regression to identify independent predictors.

Results

Clinical success was achieved in 54 patients (71.1%). There were no statistically significant differences in baseline clinical, MRI, or urodynamic parameters between the success and failure groups. Prostate volume was higher in the failure group (103.3 mL vs 88.7 mL), approaching statistical significance (p = 0.093). At three months, patients with successful outcomes demonstrated significant improvements in IPSS (12.1 vs 21.8), Qmax (13.8 vs 10.1 mL/s), and post-void residual volume (51.9 vs 107.7 mL) (all p < 0.001). On multivariate analysis, prostate volume showed a borderline negative association with clinical success (β = −0.019, p = 0.051), while other variables, including transition zone index and BOOI, were not significant predictors.

Conclusion

PAE is an effective treatment for LUTS due to BPH, with clinical success achieved in the majority of patients. Pre-procedural MRI and urodynamic parameters demonstrated limited value in independently predicting outcomes. Prostate volume may have a potential predictive role; however, its association remains inconsistent. These findings suggest that multimodal and dynamic post-procedural factors may be more relevant in predicting treatment success.

## Introduction

Benign prostatic hyperplasia (BPH) is one of the most common conditions in aging men and a serious contributor to lower urinary tract symptoms (LUTS), having serious adverse effects on the quality of life. As people get older, BPH becomes more common, with over 50 percent of men being older than 60 years, with symptoms ranging from mild urinary problems to severe acute urinary retention and related problems [1,2]. The nature of the disease and the morbidity associated with it are progressive, and this puts a significant load on the healthcare systems all over the world [3].

Treatment of symptomatic BPH involves drug treatment and surgery. Alpha-blockers and 5-alpha reductase inhibitors remain first-line medical treatments, but long-term effectiveness may be limited by poor symptom control and adverse effects [4]. Surgery procedures like transurethral resection of the prostate (TURP) have been the gold standard in treating moderate and severe diseases, but are characterized by perioperative complications such as bleeding, sexual dysfunction, and problems with the urethra [5]. These restrictions have led to the creation of minimally invasive substitutes.

Prostate artery embolization (PAE) is now a safe and effective minimally invasive method of treating LUTS secondary to BPH. In the last ten years, these results have become more clinically evident, showing substantial changes in symptom scores, urinary flow parameters, and quality of life after PAE, with a favorable safety profile [6]. Proclaimed clinical success rates exceed 80% after short-term follow-up; nevertheless, a significant number of patients report less-than-optimal response or clinical failure [7]. Clinical success following PAE has shown variability, prompting growing interest in identifying predictors of clinical success. Several clinical and procedural variables have been studied, including baseline symptom levels, prostate size, and comorbidity, with results remaining inconclusive [7,8]. Such discrepancies indicate the need for more accurate and objective predictors of pre-procedures to select patients.

MRI is a significant imaging modality used in patient assessment for PAE, as it allows detailed evaluation of prostate volume, zonal anatomy, and morphology. Some features of MRI, such as adenomatous-dominant hyperplasia and glandular morphology, have been postulated to affect treatment outcomes, but the evidence is inconclusive [9]. Besides, biomarker imaging, including prostate infarction, has also been found to correlate with success in treatment, which indicates that MRI can be used to predict outcome [10]. Urodynamic studies provide an adjunct functional measure that determines bladder outlet obstruction and detrusor function by analyzing pressure and flow. Maximum urinary flow rate (Qmax) and parameters such as detrusor pressure provide objective measures of disease severity and response to the intervention. Although PAE has been shown to enhance urodynamic parameters, its predictive value for treatment success has not been sufficiently investigated [11].

Since BPH is a structural and functional issue, a combined approach of anatomical analysis and functional assessment could provide a more in-depth understanding of disease behavior and treatment response. These parameters, however, have been examined by the existing literature at face value. Thus, the purpose of the current research is to examine MRI pre-procedural parameters and urodynamic outcomes to predict post-prostate artery embolization clinical success, with the aim of enhancing patient selection and clinical outcomes.

## Materials and methods

This is a retrospective observational study conducted at Fatima Bashir Hospital, Pakistan, with approval from the Institutional Review Board (IRB Approval No: IRB# FBH -96-(08)). It involved a sample of male patients who had PAE because of moderate to severe LUTS during a specific period of time and were diagnosed with BPH. Hospital electronic medical records and radiology databases were searched to retrieve patient data.

The anticipated proportion of clinical success after PAE was used to determine the sample size. Other multicenter studies conducted in the past have reported clinical success rates of about 80-85% at short-term follow-up [1]. The minimum required sample size was then obtained from the single proportions formula with the following parameters: proportion (p = 0.85), confidence level (95%), and margin of error (8%): n = Z² × p × (1 − p) / d², where Z = 1.96 (95% confidence interval), p = 0.85, and d = 0.08. This yielded a minimum sample size of approximately 77 patients. To account for incomplete data and potential exclusions, a slightly larger sample was considered appropriate.

Patients aged ≥50 years with symptomatic “BPH (defined as International Prostate Symptom Score (IPSS) ≥8)” who underwent pre-procedural MRI and urodynamic evaluation prior to PAE were included. Only patients with complete baseline and three-month follow-up data were analyzed. Patients with known prostate cancer, prior prostate surgery, neurogenic bladder, urethral stricture disease, or incomplete records were excluded. The inclusion and exclusion criteria applied in this study are summarized in Table 1.

**Table 1:** Inclusion and exclusion criteria BPH = Benign Prostatic Hyperplasia; IPSS = International Prostate Symptom Score; PAE = Prostate Artery Embolization; MRI = Magnetic Resonance Imaging.

Inclusion Criteria	Exclusion Criteria
Male patients aged ≥50 years	Known prostate malignancy
Symptomatic BPH (IPSS ≥8)	Prior prostate surgery
Undergoing prostate artery embolization (PAE)	Neurogenic bladder dysfunction
Availability of pre-procedural MRI	Urethral stricture disease
Availability of urodynamic evaluation	Incomplete clinical, imaging, or follow-up data
Complete 3-month follow-up data	

Baseline clinical variables included age, comorbid conditions, IPSS, quality of life (QoL) score, maximum urinary flow rate (Qmax), and post-void residual urine (PVR). Pre-procedural MRI parameters included prostate volume, transition zone index, intravesical prostatic protrusion, and the presence of adenomatous-dominant hyperplasia, assessed by an experienced radiologist. Urodynamic parameters included Qmax, detrusor pressure at maximum flow (PdetQmax), and the bladder outlet obstruction index (BOOI), calculated as: BOOI = PdetQmax − 2 × Qmax [12].

Pre-procedural magnetic resonance imaging (MRI) was performed using a 1.5-Tesla scanner with pelvic phased-array coils. Standard imaging sequences included axial and sagittal T2-weighted images, diffusion-weighted imaging, and contrast-enhanced sequences. Prostate volume was calculated using the ellipsoid formula (length × width × height × 0.52), while transition zone index was derived by dividing transition zone volume by total prostate volume. Intravesical prostatic protrusion was measured in the sagittal plane as the vertical protrusion of the prostate into the bladder lumen. Urodynamic studies were performed according to International Continence Society standards using multichannel pressure-flow studies with normal saline at a filling rate of 30-50 mL/min. The bladder outlet obstruction index (BOOI) was calculated using the Abrams-Griffiths formula (BOOI = PdetQmax − 2 × Qmax). During prostate artery embolization, selective catheterization was performed using a 2.0-2.4 Fr microcatheter, and embolization was achieved with calibrated tris-acryl gelatin microspheres (300-500 μm) until near-stasis of arterial flow was observed.

All patients underwent prostate artery embolization performed by an experienced interventional radiologist using a standardized technique under local anesthesia. Selective catheterization of the prostatic arteries was achieved via femoral or radial access, followed by embolization with calibrated microspheres until near-stasis was observed. Technical success was defined as successful embolization of at least one prostatic artery. Patients were followed up at three months post-procedure. Clinical evaluation included reassessment of IPSS, QoL score, Qmax, and PVR. Clinical success was defined as a reduction in IPSS of ≥25% from baseline and/or an improvement in Qmax of ≥2 mL/s, consistent with previously reported outcome measures in PAE studies [1,2]. Patients who did not meet these criteria were categorized as having clinical failure.

SPSS software, version 26.0, was used to analyze data. Mean ± standard deviation or median with interquartile range were used to report quantitative variables based on the data distribution, whereas frequencies and percentages were used to report qualitative variables. There were successful and unsuccessful outcome groups that were stratified into patients. The comparison of continuous variables was done with an independent-samples t-test or a Mann-Whitney U test, and that of categorical variables was done with a Chi-square test or Fisher’s exact test. Pearson or Spearman coefficients were also used to assess correlations. The analysis was performed using multivariate logistic regression to determine independent predictors of clinical success. A p-value < 0.05 was considered significant. 

## Results

A total of 76 patients were included in the final analysis. The mean age of the study population was 66.5 ± 7.3 years. Clinical success at three-month follow-up was achieved in 54 patients (71.1%), while 22 patients (28.9%) were classified as having clinical failure. Baseline demographic, clinical, MRI, and urodynamic characteristics are presented in Table 2. There was no statistically significant difference in age between patients with successful and unsuccessful outcomes (66.3 vs 67.0 years, p = 0.655). Similarly, baseline symptom severity, assessed using the International Prostate Symptom Score (IPSS), did not differ significantly between groups (21.7 vs 23.4, p = 0.219). Baseline functional parameters, including maximum urinary flow rate (Qmax) and post-void residual volume (PVR), were also comparable between the two groups, with mean Qmax values of 8.60 mL/s and 8.97 mL/s (p = 0.527), and mean PVR values of 116.4 mL and 122.2 mL (p = 0.748) in the success and failure groups, respectively.

**Table 2:** Baseline characteristics of study population IPSS = International Prostate Symptom Score; Qmax = Maximum Urinary Flow Rate; PVR = Post-Void Residual Volume; BPH = Benign Prostatic Hyperplasia; PdetQmax = Detrusor Pressure at Maximum Flow; BOOI = Bladder Outlet Obstruction Index.

Variable	Clinical Success (n = 54)	Clinical Failure (n = 22)	p-value
Age (years)	66.3 ± 7.1	67.0 ± 7.8	0.655
IPSS (baseline)	21.7 ± 4.8	23.4 ± 5.2	0.219
Qmax (mL/s)	8.60 ± 2.0	8.97 ± 2.1	0.527
PVR (mL)	116.4 ± 58.2	122.2 ± 61.5	0.748
Prostate Volume (mL)	88.7 ± 28.5	103.3 ± 32.7	0.093
Transition Zone Index	0.56 ± 0.09	0.54 ± 0.10	0.412
Adenomatous BPH (%)	42.6%	45.5%	0.812
Intravesical Protrusion (%)	46.3%	36.4%	0.437
PdetQmax (cmH₂O)	75.9 ± 18.6	74.7 ± 19.2	0.781
BOOI	58.3 ± 15.4	56.4 ± 16.1	0.689

Among categorical variables, adenomatous-dominant benign prostatic hyperplasia showed no significant association with clinical outcome (42.6% vs 45.5%; χ²(1) = 0.06, p = 0.812, Cramér’s V = 0.03). Similarly, intravesical prostatic protrusion was not significantly associated with outcome (46.3% vs 36.4%; χ²(1) = 0.61, p = 0.437, Cramér’s V = 0.09). The presence of comorbidities also did not show a significant association with clinical success (χ²(1) ≈ 0.07, p = 0.784, Cramér’s V ≈ 0.03).

Among MRI-derived parameters, prostate volume was higher in patients with unsuccessful outcomes (103.3 mL) compared to those with successful outcomes (88.7 mL), approaching statistical significance (p = 0.093). However, the transition zone index did not differ significantly between groups (p = 0.412). Urodynamic parameters, including detrusor pressure at maximum flow (PdetQmax) and bladder outlet obstruction index (BOOI), were also comparable between groups, with no statistically significant differences (p = 0.781 and p = 0.689, respectively).

At three-month follow-up, patients who achieved clinical success demonstrated significant improvement in symptom and functional parameters (Table 3). The mean IPSS was significantly lower in the success group compared to the failure group (12.1 vs 21.8, p < 0.001). Similarly, Qmax improved significantly in the success group (13.8 mL/s) compared to the failure group (10.1 mL/s, p < 0.001). Post-void residual volume was also significantly lower in the success group (51.9 mL vs 107.7 mL, p < 0.001). Quality-of-life scores showed parallel improvement (2.57 vs 3.82, p < 0.001).

**Table 3:** Comparison of post-procedural outcomes at three months IPSS = International Prostate Symptom Score; QoL = Quality of Life; Qmax = Maximum Urinary Flow Rate; PVR = Post-Void Residual Volume.

Variable	Clinical Success	Clinical Failure	p-value
IPSS (3 months)	12.1 ± 4.3	21.8 ± 5.1	<0.001
QoL Score	2.57 ± 1.2	3.82 ± 1.1	<0.001
Qmax (mL/s)	13.8 ± 3.5	10.1 ± 2.8	<0.001
PVR (mL)	51.9 ± 40.2	107.7 ± 55.8	<0.001

Multivariate logistic regression analysis was performed to identify independent predictors of clinical success (Table 4). Prostate volume demonstrated a borderline statistically significant negative association with clinical success (β = −0.019, odds ratio (OR) = 0.98, 95% confidence interval (CI): 0.96-1.00, p = 0.051), suggesting that larger prostate size may be associated with a reduced likelihood of a favorable outcome. Other variables, including age (p = 0.645), comorbidities (p = 0.784), baseline PVR (p = 0.425), transition zone index (p = 0.634), adenomatous-dominant hyperplasia (p = 0.798), and BOOI (p = 0.818), were not identified as independent predictors.

**Table 4:** Multivariate logistic regression analysis for predictors of clinical success OR = Odds Ratio; CI = Confidence Interval; PVR = Post-Void Residual Volume; BPH = Benign Prostatic Hyperplasia; BOOI = Bladder Outlet Obstruction Index.

Variable	β Coefficient	Odds Ratio (OR)	95% CI	p-value
Age	-0.017	0.98	0.92 – 1.06	0.645
Comorbidities	-0.145	0.87	0.31 – 2.44	0.784
PVR (baseline)	-0.003	1.00	0.99 – 1.01	0.425
Prostate Volume	-0.019	0.98	0.96 – 1.00	0.051
Transition Zone Index	1.328	3.78	0.02 – 899.59	0.634
Adenomatous BPH	-0.144	0.87	0.29 – 2.60	0.798
BOOI	0.003	1.00	0.98 – 1.03	0.818

In general, in most of the patients, clinical success was reached after prostate artery embolization. Most of the clinical, imaging, and urodynamic parameters at baseline showed no significant relationship with outcome, although prostate volume showed the possibility of making a prognosis, and a larger gland size favored a worse response. Symptom scores, urinary flow, and residual volume showed significantly better outcomes among patients with successful outcomes after procedures.

## Discussion

The present study evaluated the role of pre-procedural MRI parameters and urodynamic findings in predicting clinical success following prostate artery embolization (PAE). Clinical success was achieved in 71.1% of patients, comparable to previously reported rates of 70% to 85% in short-term follow-up studies [13,14]. This supports the growing body of evidence that PAE is an effective minimally invasive treatment for symptomatic benign prostatic hyperplasia (BPH), although a subset of patients continues to demonstrate suboptimal response. A key finding of this study was the absence of statistically significant independent predictors of clinical success on multivariate analysis, with the exception of a borderline association with prostate volume. This observation aligns with existing literature, which consistently reports heterogeneity and inconsistency in predictive factors for PAE outcomes. Several studies have attempted to identify reliable predictors; however, results remain conflicting, reflecting the multifactorial nature of treatment response [15,16].

Prostate volume has been one of the most frequently studied variables in this context. In the present study, larger prostate volume demonstrated a borderline negative association with clinical success (p = 0.051), suggesting a potential trend toward reduced efficacy in larger glands. However, the literature remains inconclusive. While some studies have reported improved outcomes in patients with larger prostates, possibly due to greater ischemic volume reduction [17], others have found no significant relationship between prostate size and clinical outcomes [18]. The STREAM trial similarly demonstrated that baseline prostate volume was not a reliable predictor of long-term symptom improvement [8]. These discrepancies highlight the complex interplay between gland size, vascular supply, and embolization efficacy, suggesting that prostate volume alone may be insufficient as a predictive marker.

MRI-derived parameters, including transition zone index and adenomatous-dominant hyperplasia, did not demonstrate a significant association with clinical outcomes in this study. This finding is consistent with recent evidence suggesting that conventional MRI parameters may have limited predictive value. Boschheidgen et al. reported that although certain morphological features such as adenomatous-dominant hyperplasia may correlate with volume reduction and symptom improvement, their predictive value remains inconsistent [6]. Similarly, Martin et al. found that advanced imaging approaches, including radiomics, provided limited additional benefit in predicting outcomes, with only weak associations observed in selected models [7]. These findings indicate that while MRI is invaluable for anatomical assessment and procedural planning, its role as a standalone prognostic tool remains limited.

Urodynamic parameters, including bladder outlet obstruction index (BOOI) and detrusor pressure, were also not significantly associated with clinical success in the present study. Although urodynamic studies provide an objective assessment of bladder outlet obstruction, their role in predicting response to PAE is not well established. Previous studies have demonstrated significant improvement in urodynamic parameters following PAE; however, baseline values have not consistently predicted treatment outcomes [19]. This suggests that while urodynamics are useful for diagnostic evaluation, they may not adequately capture the vascular and tissue-level changes induced by embolization.

Another important observation in this study was the lack of significant association between baseline symptom severity and treatment outcome. This finding is in agreement with prior studies demonstrating that baseline IPSS does not reliably predict response to PAE [20]. However, post-procedural improvement in symptom scores remains a robust indicator of treatment success, as also observed in this study. The absence of strong independent predictors in this study reflects a broader trend in the literature, where no single clinical, imaging, or functional parameter has consistently demonstrated reliable prognostic value. Fei Sun et al. emphasized that predictive factors in PAE can be broadly categorized into baseline, early post-procedural, and follow-up variables, with early imaging biomarkers, such as infarction volume, showing greater promise than baseline characteristics [14]. Similarly, studies evaluating post-procedural MRI findings have demonstrated that the extent of prostatic infarction correlates more strongly with clinical outcomes than pre-procedural parameters [21]. This suggests that dynamic treatment-related factors may play a more critical role in determining outcome than baseline patient characteristics.

The findings of this study underscore the importance of a comprehensive and multimodal approach to patient evaluation. Given that BPH is a multifactorial disease involving anatomical enlargement, functional obstruction, and vascular components, it is unlikely that a single parameter can accurately predict treatment response. Instead, a combination of clinical, imaging, and procedural factors, potentially integrated through predictive models or machine learning approaches, may provide a more accurate framework for outcome prediction [20].

This study has several limitations that should be acknowledged. The retrospective design introduces potential selection bias, and the relatively modest sample size may limit statistical power, particularly for detecting subtle associations. The wide confidence intervals observed for certain variables, such as the transition zone index, further reflect variability within the dataset. Additionally, only short-term outcomes at three months were evaluated, whereas longer follow-up may provide a more robust assessment of treatment durability. Despite these limitations, the study provides valuable insights into real-world predictors of PAE outcomes and reinforces findings reported in contemporary literature.

## Conclusions

In conclusion, the present study demonstrates that although prostate artery embolization is effective in most patients, pre-procedural MRI and urodynamic parameters have limited value in independently predicting clinical success. Prostate volume may be associated with outcomes; however, this relationship remains inconsistent. These findings highlight the need for further research focusing on integrated predictive models and dynamic post-procedural markers to improve patient selection and optimize clinical outcomes.
